# ExpoSeq: simplified analysis of high-throughput sequencing data from antibody discovery campaigns

**DOI:** 10.1093/bioadv/vbae020

**Published:** 2024-02-10

**Authors:** Christoffer V Sørensen, Nils Hofmann, Puneet Rawat, Frederik V Sørensen, Anne Ljungars, Victor Greiff, Andreas H Laustsen, Timothy P Jenkins

**Affiliations:** Department of Biotechnology and Biomedicine, Technical University of Denmark, DK-2800 Kongens Lyngby, Denmark; Department of Biotechnology and Biomedicine, Technical University of Denmark, DK-2800 Kongens Lyngby, Denmark; Department of Immunology, University of Oslo and Oslo University Hospital, NO-0316 Oslo, Norway; Bornerups A/S, DK-7700 Thisted, Denmark; Department of Biotechnology and Biomedicine, Technical University of Denmark, DK-2800 Kongens Lyngby, Denmark; Department of Immunology, University of Oslo and Oslo University Hospital, NO-0316 Oslo, Norway; Department of Biotechnology and Biomedicine, Technical University of Denmark, DK-2800 Kongens Lyngby, Denmark; Department of Biotechnology and Biomedicine, Technical University of Denmark, DK-2800 Kongens Lyngby, Denmark

## Abstract

**Summary:**

High-throughput sequencing (HTS) offers a modern, fast, and explorative solution to unveil the full potential of display techniques, like antibody phage display, in molecular biology. However, a significant challenge lies in the processing and analysis of such data. Furthermore, there is a notable absence of open-access user-friendly software tools that can be utilized by scientists lacking programming expertise. Here, we present ExpoSeq as an easy-to-use tool to explore, process, and visualize HTS data from antibody discovery campaigns like an expert while only requiring a beginner’s knowledge.

**Availability and implementation:**

The pipeline is distributed via GitHub and PyPI, and it can either be installed as a package with pip or the user can choose to clone the repository.

## 1 Introduction

Monoclonal antibodies have revolutionized the treatment of various diseases, including cancer and autoimmune diseases (Kaplon *et al.* 2020). For the discovery of these antibodies, *in vitro* display technologies have played an important role (Bradbury *et al.* 2011, Bazan *et al.* 2012, Frenzel *et al.* 2016, Laustsen *et al.* 2021). One of the major advantages of *in vitro* display technologies is their utility for rapidly screening and/or selecting antibodies from very large libraries (Ledsgaard *et al.* 2018). This enables the identification of highly specific, high-affinity antibodies to a wide range of target antigens, including small molecules, peptides, proteins, and cells regardless of their immunogenicity and toxicity (Bradbury *et al.* 2011). One of the most commonly used *in vitro* display methods is antibody phage display technology, which involves biopanning of an antibody-displaying phage library against a target, upon which typically only a relatively small subset of the selected binders are picked for screening, (Sanger) sequencing, and functional assays (Ledsgaard *et al.* 2018, Alfaleh *et al.* 2020). While antibody phage display technology has been utilized to discover several successful therapeutic antibodies, such as one of the top-selling drugs, adalimumab (Ecker *et al.* 2015, Grilo and Mantalaris 2019), recent studies suggest that to truly unlock the full potential of display technologies, high-throughput sequencing (HTS) is key (Domina *et al.* 2014, Liu *et al.* 2015, Yang *et al.* 2017, Ljungars *et al.* 2019, Mattsson *et al.* 2023). The reason for this is that HTS allows for the screening and analysis of the entire pool of antibodies during the biopanning process, rather than the assessment and characterization of only a small subset of the most abundant clones. This may allow for the identification of rare clones in the antibody pools that might otherwise have been missed (Ljungars *et al.* 2019). Further, the use of HTS might provide large and high quality antibody datasets, including sequences of antibodies with various binding characteristics, that to date remain scarce, yet essential, as a resource in the global drive towards the prediction of protein-protein interactions using sophisticated machine learning approaches (Laustsen *et al.* 2021, Akbar *et al.* 2022). However, while the use of HTS presents a plethora of opportunities, it also comes with substantial hurdles and requires advanced analytics and the ability to process complex datasets. This can be a challenging and time-consuming process, often requiring specialized computational skills to effectively analyse and utilize the data.

**Figure 1.**
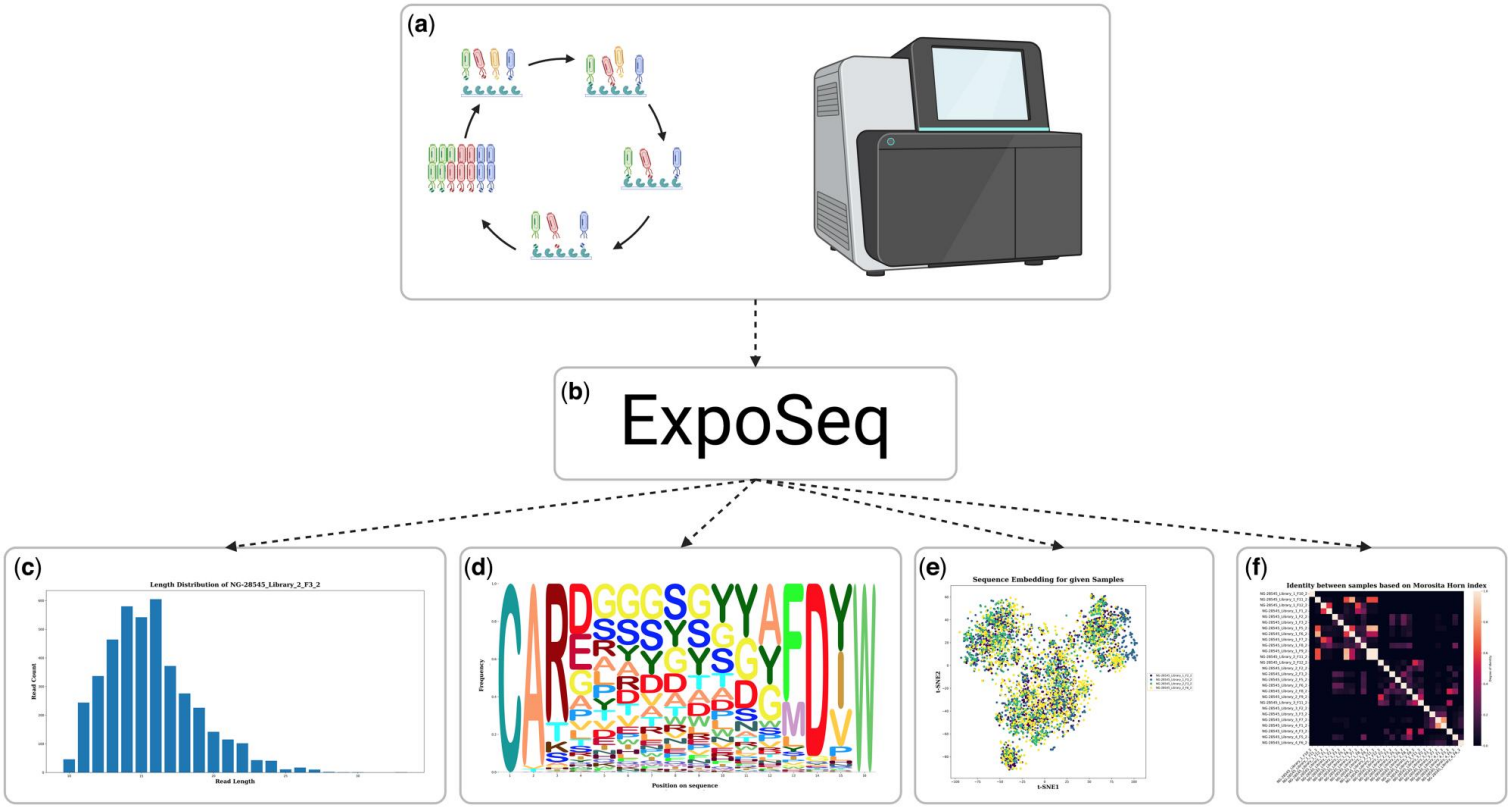
Schematic overview of the functionality of ExpoSeq: (a) HTS of repertoires of discovered antibodies. (b) The HTS fastq files and binding data (if available) is uploaded to ExpoSeq, which processes the data and generates quality control and analytical plots, either automatically or user-guided. (c) Example plot: length distribution of the reads for a given specific sample. (d) Example plot: sequence logo plot for a given sequence length of a specific sample. (e) Example plot: T-SNE clustering of the sequence embedding for three different samples offering an overview of the sequence similarity. (f) Example plot: matrix for all sequenced panning rounds showing overlap between the samples based on Morosita-Horn index. The figure is created with BioRender.com.

Here, we introduce ExpoSeq, a user-friendly Python-based pipeline designed to streamline the analysis of HTS data from extensive antibody pools (Fig. 1). With the ExpoSeq pipeline, we hope to make the analysis of HTS data from antibody discovery campaigns more accessible to non-bioinformatician experts and provide a fast and modular tool that facilitates rapid data interpretation and visualization. In comparison to existing tools, such as Immunarch, Vdjtools, Immcantation, and Immuno Data Analyzer, which have already simplified HTS analysis for antibody immune repertoires, ExpoSeq differs in two key aspects. Firstly, ExpoSeq is tailored specifically for analysing HTS data from *in vitro* antibody discovery campaigns. This customization includes specialized features such as sample-to-sample similarity heat maps and the capability to analyse all or individual complementarity-determining regions (CDRs), rather than solely the heavy-chain CDR3 (HCDR3). Most notably, ExpoSeq allows for the connection of antibody binding data to HTS results. This integration enables researchers to identify sequence motifs associated with certain binding properties of the antibody, facilitating a better understanding of the molecular basis of antibody-target interactions. Moreover, in cases where only a subset of antibodies has undergone binding analysis, linking binding data to HTS helps identify closely related antibodies for further testing or consideration to expand the number of potential lead antibodies. Secondly, ExpoSeq offers an intuitive workflow, featuring prompts that seamlessly guide the user through each stage of the pipeline. This workflow has been meticulously condensed and simplified to maximize user-friendliness. As a result, tasks such as pipeline installation, initiation, upload of sequences, antibody binding data import, and all subsequent plot and data analysis steps have been streamlined into an efficient process involving the answering of just a few prompts. These prompts primarily entail selecting files, pressing enter, or responding with a simple yes or no. Additionally, ExpoSeq can function with nearly all the analytical presets offered by MiXCR, and the pipeline also allows the MiXCR analysis to be carried out on external high-performance computing systems to enable researchers with such resources to carry out the analysis faster.

## 2 ExpoSeq description

### 2.1 Functionalities

Initially, the user is guided through the preprocessing of their data (in fastq format) using the MiXCR software to reduce noise, isolate the CDRs of interest, count the reads, and sort the reads (Bolotin *et al.* 2015). In the example showcased here, we used MiXCR (4.2.0) with the alignment setting ‘milab-human-tcr-dna-multiplex-cdr3’, but other built-in MiXCR presets are also usable, which are listed prior to the processing of the fastq files. ExpoSeq implements two additional data trimming steps, which involve the removal of nucleotide reads that are not divisible by 3, have a lower clone count than 3, or are <18 nucleotides in length (these parameters can all be changed in the pipeline after the processing has been terminated). Following this initial data processing, the automatic analysis is initiated, creating a table containing all relevant data followed by generating the different plots offered by the pipeline. The constructed table contains all relevant data, including read count, read fraction, sequences, experiment name, etc., which can be used after the automation to individualize the visualizations and carry out a customized analysis. Furthermore, the software offers a high degree of flexibility with its ability to switch to different regions on the heavy chain or adding binding data to a subset of your sequences, if such data is available. Lastly, a report can be generated using the software. However, the reports are not required for using ExpoSeq and are additions that aid in the data exploration. In the following, a few functions offered by ExpoSeq for the analysis of HTS data from antibody discovery campaigns are presented.

### 2.2 Sequencing QC

To assess sequence quality and identity, a series of plots can be generated to identify potential issues with the sequencing data, such as insufficient sequencing depth or potential contamination; this helps ensure the accuracy and reliability of experimental results. In addition, the quality assessment can provide valuable information for troubleshooting and optimization of experimental protocols.

### 2.3 Visualization of clone count alignment success

The first QC plot shows the number of total reads carried out per sample and how many of these reads were successfully aligned using MiXCR (Supplementary Fig. S1). From this, the user can see whether any samples have been underrepresented in the sequencing, or whether MiXCR has removed a high percentage of sequences due to alignment errors, such as absence of immunoglobulin (Ig) sequence, low total alignment score, or absence of particular gene segments amongst others.

### 2.4 Rarefaction curves

Rarefaction curves can be generated to assess if the sequencing depth is sufficient to cover the majority of antibody sequences in the tested samples (Rodriguez-R and Konstantinidis 2014). ExpoSeq achieves this by randomly dividing a sample into 100 bins and additively counting the number of unique sequences in each bin. This results in 100 data points that present an increasing number of unique sequences. If these points form a diagonal line, it indicates that deeper sequencing is necessary to cover the majority of unique sequences. In contrast, if the line plateaus as it progresses through the bins, it suggests that sufficient sequencing depth has been achieved, because the number of unique sequences encountered decreases gradually (Supplementary Fig. S2).

### 2.5 Diversity

The diversity of the amino acid sequences within one sample can provide a measurement for the quality of the library diversity (Ledsgaard *et al.* 2022) or the success of subsequent display rounds. ExpoSeq offers the option of visualizing this based on the Shannon (Shannon 1948) (Supplementary Fig. S3) and Inverse Simpson index (Simpson 1949) (Supplementary Fig. S4) where the probability for these measurements is represented by the clone fraction of each individual sequence. The output is represented as a barplot where high values in comparison to lower values of successive rounds could indicate enrichment of certain sequences.

### 2.6 Comparing the sequence outputs

Upon confirmation of the initial QC steps, ExpoSeq makes it possible to study the sequence outputs using three different methods.

#### 2.6.1 Heatmaps

Heatmaps are useful tools for assessing the similarity between samples and can serve as both an extended quality control and sample analysis tool. ExpoSeq offers four methods for constructing these heatmaps: Relative index, Morisita-Horn index, Jaccard index, and Sørensen-Dice index. While the visualization and algorithm remain the same, the calculation of the indexes differs between the methods. For example, the relative index (Supplementary Fig. S5) compares the number of identical sequences between two samples without considering how many times each sequence was present, whereas the Morisita-Horn index (Supplementary Fig. S6) takes the sequence count into consideration.

#### 2.6.2 CDR length distribution

To observe trends in the length of the CDRs (HCDR3 in our example), length distribution plots can be used. Using ExpoSeq, these plots can be constructed on a single-sample basis or for multiple datasets with individual samples as subplots (Supplementary Fig. S7).

#### 2.6.3 Amino acid distribution

ExpoSeq offers two methods for visualizing the amino acid (AA) composition of a specific region of interest, such as the HCDR3. To ensure accurate comparisons, it is recommended to only compare sequences of identical length. This is particularly important when comparing the HCDR3s of antibodies, which can vary greatly in length but generally have less variation in the beginning and end. Comparing HCDR3s of different lengths can lead to inaccurate results, as the ‘end-position’ AAs will appear overrepresented due to the shorter sequences ending in the middle of longer sequences when compared from beginning to end. The two ways of comparing AA composition in ExpoSeq are with a sequence logo plot using logomaker (Tareen and Kinney 2020) (Supplementary Fig. S8) and a stacked bar plot (Supplementary Fig. S9).

### 2.7 Sequence clustering

To dissect sequence similarities within a dataset or between datasets, sequence similarity clustering tools were implemented in the pipeline. This can help to elucidate enrichment of sequences with similar characteristics, which can be used for selecting or deselecting specific groups of discovered antibodies.

### 2.8 Clustering based on Levenshtein distance

Levenshtein Distance (LD) is used to measure the substitutions, additions, or deletions necessary to make two different text sequences identical (Miho *et al.* 2019): e.g. there is an LD of 2 between ‘CAT’ and ‘BAD’ since both the C and T in ‘CAT’ need to be changed to make the words identical, whereas ‘CAT’ and ‘CATS’ have an LD of 1, since only an S needs to be added to make the words identical. This theory can be applied to AA sequences, as well and can be used as a method to cluster similar HCDR3 sequences. In ExpoSeq, sequences can be clustered in a dendrogram based on their LD to other sequences (Supplementary Fig. S10). Further, if instead of a dendrogram, a node-cluster approach is preferred by the user, this can also be carried out with connected nodes being based on different LD cutoffs (Supplementary Fig. S11).

### 2.9 Clustering based on sequence embedding

Levenshtein Distances have the limitation that they do not address the different properties of the AAs, nor can they derive patterns from multiple sequences. Therefore, a plot type was created, which takes the whole sequence relation into account to enable a more extensive analysis of the sequences. To be able to have the sequences as vectors, the Sequence Graph Transform (SGT) embedding was applied. This embedding does not capture any chemical properties of the amino acid strand, but instead tries to recognize the characteristic relative position of letters within a sequence that enables an identification of patterns between sequences of different lengths. This addresses the variability of the HCDR3 sequences and enables sequence similarity analysis (Ranjan *et al.* 2016). Besides, the user can also choose between models from Rostlab, which were trained on millions of protein sequences. The embedding results in a multidimensional output, which is reduced to two dimensions using Principal Component Analysis (PCA) and t-distributed Stochastic Neighbour Embedding (t-SNE) (Van der Maaten and Hinton 2008) (Supplementary Fig. S12).

### 2.10 Combining binding/affinity data with clustering

One of the key advantages of combining HTS approaches with *in vitro* antibody discovery techniques is the ability to identify new and promising antibodies that may have been previously overlooked. To aid in this effort, ExpoSeq allows the user to upload binding data from immunoassays for either a subset or all of the antibodies included in the analysis. This data can then be overlayed and integrated with clustering approaches to identify sequences with no prior binding data that have been identified as highly similar to high affinity binders (Supplementary Figs S13 and S14).

## 3 Conclusion

The introduction of HTS as a tool to aid the development of therapeutic antibodies using *in vitro* display methods has opened doors for more detailed, high-throughput analyses, but has simultaneously introduced challenges regarding data complexity and the need for specialized programming skills. Our newly introduced pipeline, ExpoSeq, directly addresses these challenges by simplifying the analysis of HTS data from generated pools of antibody sequences, offering a user-friendly, efficient, and adaptable tool that facilitates rapid data interpretation without the need for extensive Python knowledge. Further, looking beyond antibody discovery campaigns, the tool can also be applied to the analysis of B or T-cell repertoire sequencing data because of the uniform output of MiXCR for B-, T-cell, and IG repertoire profiling (Bolotin *et al.* 2015), which allows the user to recreate the plots in the pipeline for different types of analysis.

The functionalities of ExpoSeq range from sequencing QC and data preprocessing to visualization of the antibody pools and sequence clustering, all of which are critical steps in HTS data analysis for antibody discovery campaigns. Its comprehensive suite of visualization tools, including heatmaps, CDR length distribution plots, amino acid distribution graphs, and sequence clustering methods based on Levenshtein Distances or sequence embedding, provide an insightful and versatile toolbox for exploring data from antibody discovery campaigns. Moreover, ExpoSeq has been designed to incorporate binding and/or affinity data, enhancing its utility by linking sequence information with functional data.

## Supplementary Material

vbae020_Supplementary_Data
