# Supplementary material for: ExpoSeq: simplified analysis of high-throughput sequencing data from antibody discovery campaigns
Source: Bioinform Adv. 2024 Feb 10;4(1):vbae020. doi: 10.1093/bioadv/vbae020 (PMC10902677; doi:10.1093/bioadv/vbae020)
Supplement: vbae020_Supplementary_Data [file vbae020_supplementary_data.docx]

# Supplementary material

Contents

[Supplementary material 1](#_gjdgxs)

[Preamble 2](#_30j0zll)

Installation and getting started: 2

[Guideline for beginners 2](#_3znysh7)

[Download Python and IDE 2](#_2et92p0)

[Install ExpoSeq in IDE and analyze your data 4](#_tyjcwt)

[Supplementary Figures 8](#_3dy6vkm)

A graph of blue and orange barsDescription automatically generated 8

[How to add the binding data correctly 14](#_1t3h5sf)

[Automation of reports and interactive dashboards 15](#_cdhnppqwmnyj)

[Reproducibility 15](#_4d34og8)

[Contact and feedback 16](#_2s8eyo1)

##

## Preamble

This project was initiated to provide researchers with limited programming skills a tool to explore the powerful potential of NGS, with a focus on antibody discovery using phage display. While the tool is already promising, there is considerable scope for improvement. If you're motivated to contribute further to its development, please don't hesitate to get in touch. We're open to new collaborations, free from any biases that might hinder scientific progress or societal welfare. We warmly encourage you to share, discuss, and explore this tool with us!

## Installation and getting started:

In general, the user has two options to launch the pipeline which are designed dependent on his or her experience with the programming language python and the need for individual, customized plots. If only a general insight of the dataset is needed one can execute the script run.py and the preprocessing and generation of most of the plots is done fully automatically without any further code inputs. If one wishes to be able to change the layout of the plots or analyze, for instance the binding more extensively, it is recommended to use the Plot Manager as a toolbox to create the plots for the individual needs specifically. In the following we will continue giving examples of the pipeline executed by the plot manager.

The pipeline was designed to be used in the console in a standard IDE. We recommend PyCharm for that since this is what we have used in the following examples. If you are an experienced user you can just follow the instructions given in the [GitHub repository](https://github.com/nilshof01/ExpoSeq). Alternatively, we developed a step by step guide  which takes you through the installation and the initial launch of the pipeline. The manual starts with the installation of Python and gives a suggestion for the IDE.

## Guideline for beginners

### Download Python and IDE

Step 1: You can start by downloading the python.exe which is the programming language ExpoSeq is based on.


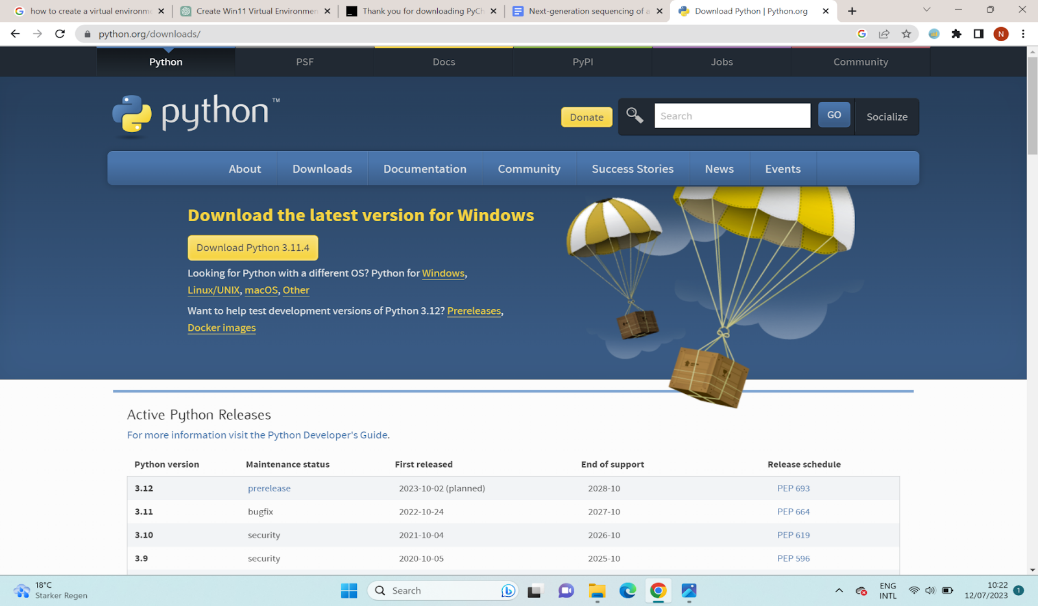


Step 2: Launch the installation and choose Customize installation. In the future, the Python version given in the picture could differ from the version you have downloaded. We suggest that you download Python 3.11.X .


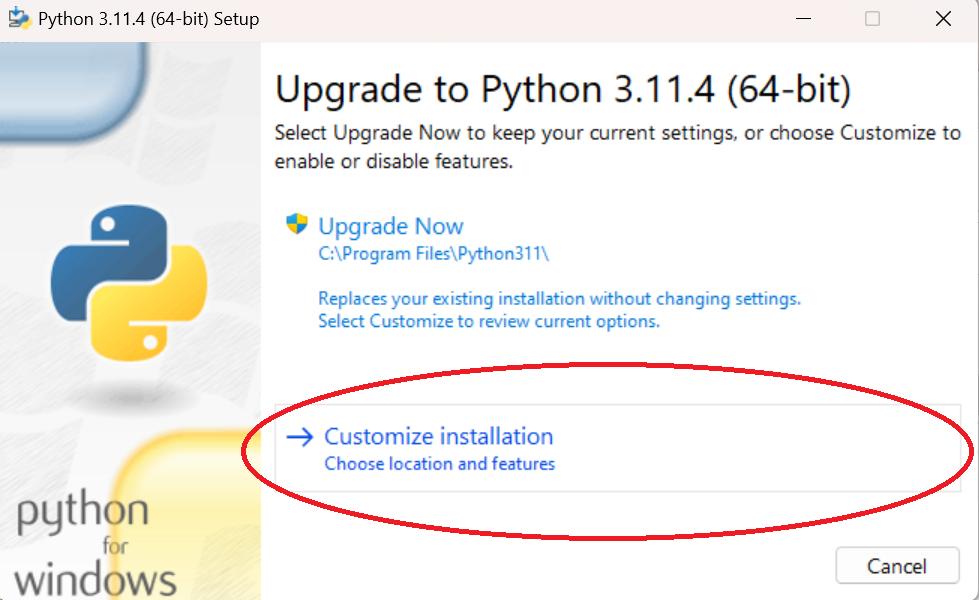


Step 3: Check if the boxes with the red circles are crossed.


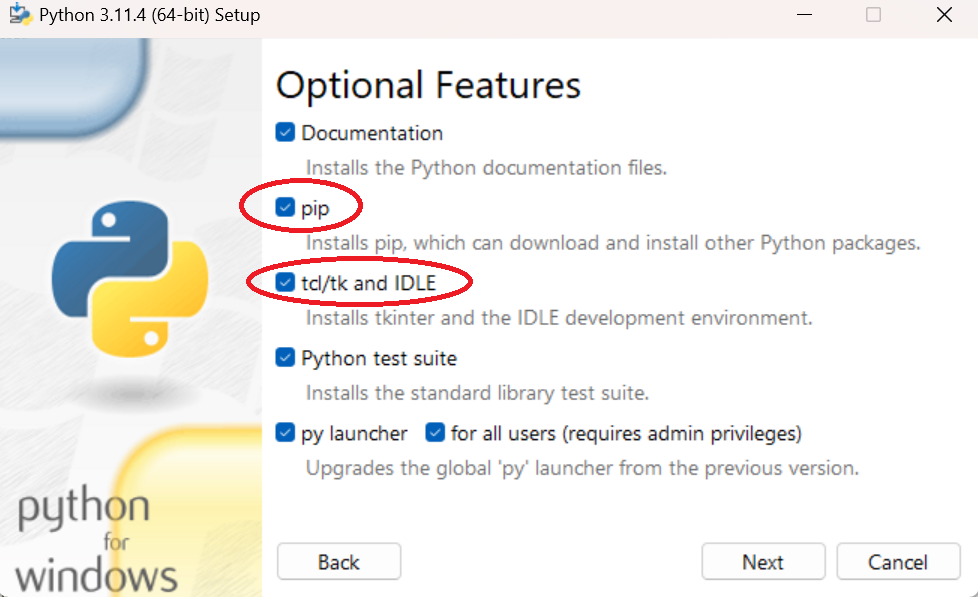


Step 4: Check whether Python will be added to your environment variables and click install.


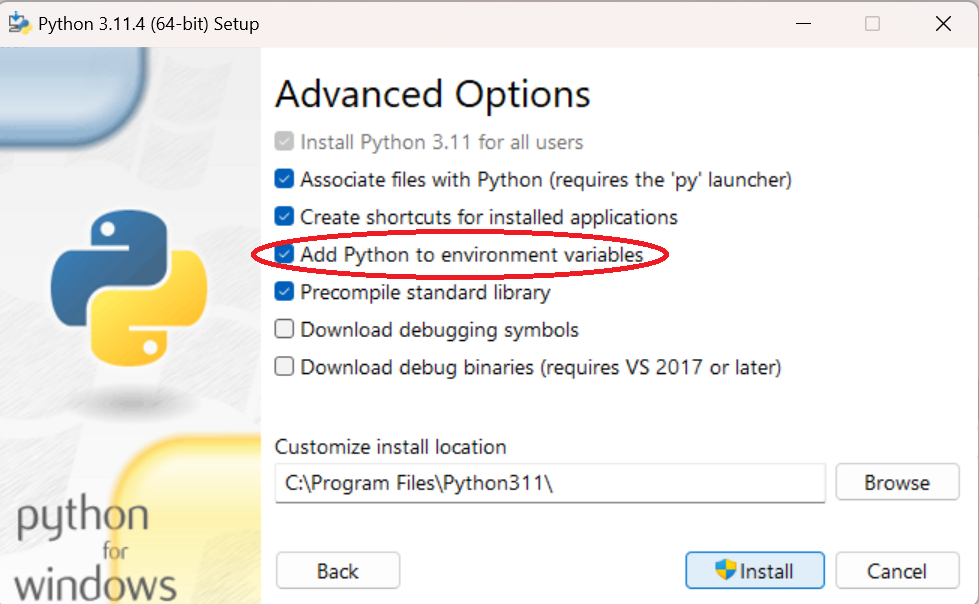


Step 5: Download [MiXCR](https://mixcr.com/mixcr/getting-started/installation/) and follow the instructions for the installation, provided on the website

Step 6: Installation of the recommended IDE: PyCharm Community Edition


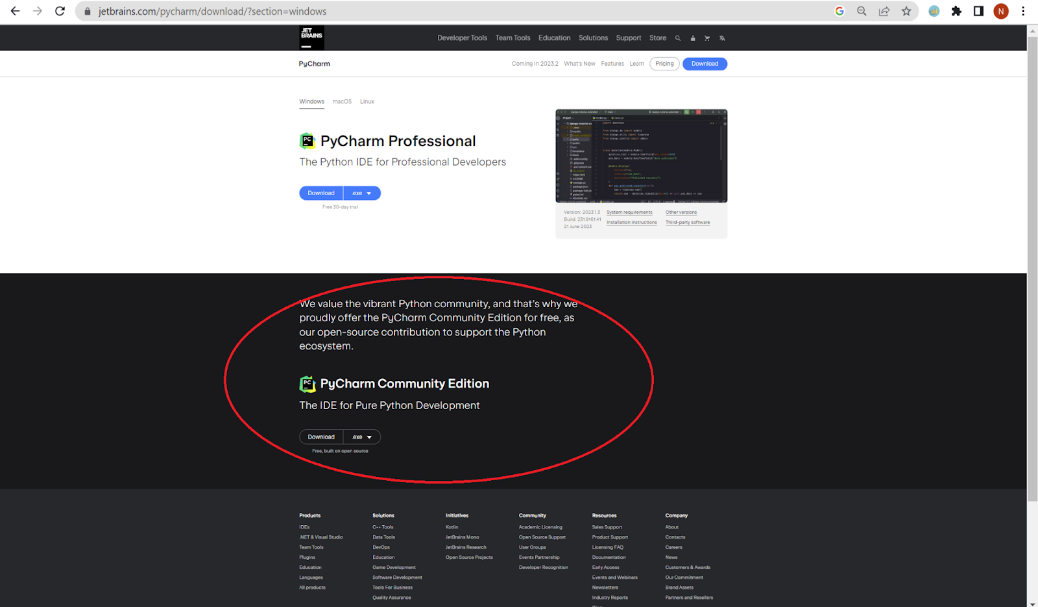


### Install ExpoSeq in IDE and analyze your data

In the following you can open PyCharm or the IDE of your preference and create a virtual environment under a new project where you will create the plots in analyze the fastq files:

Step 1: Open Pycharm and choose New Project (red circle):


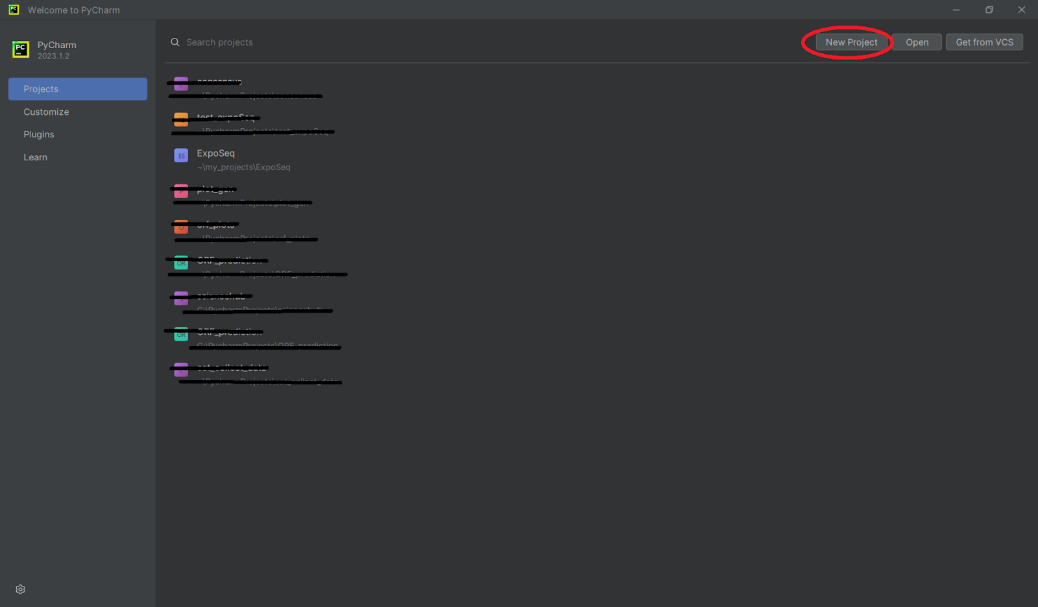


Step 2: Enter your custom project name and create a virtual environment


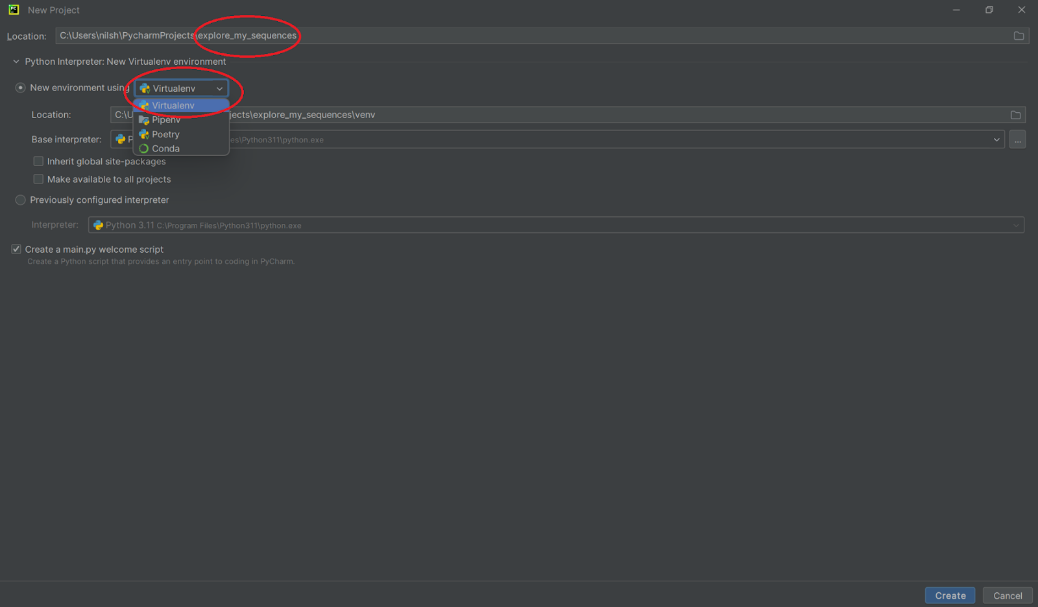


Step 3: Locate your terminal and install ExpoSeq with *pip install ExpoSeq*

Note: ExpoSeq depends on multiple other packages which will be installed automatically.


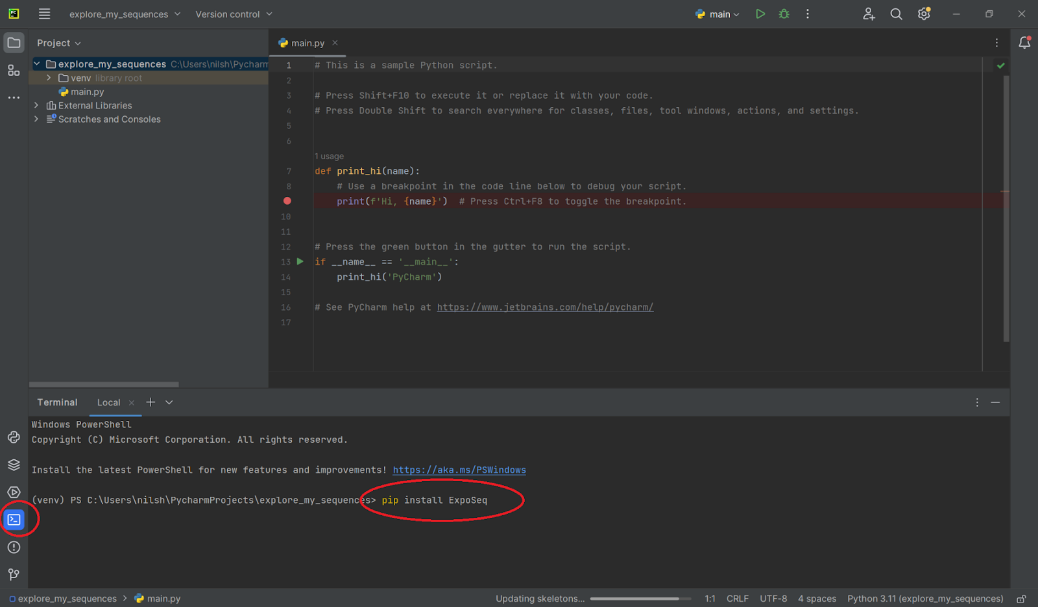


Step 4: Locate your console and import the PlotManager from ExpoSeq with *from ExpoSeq.pipeline import PlotManager*


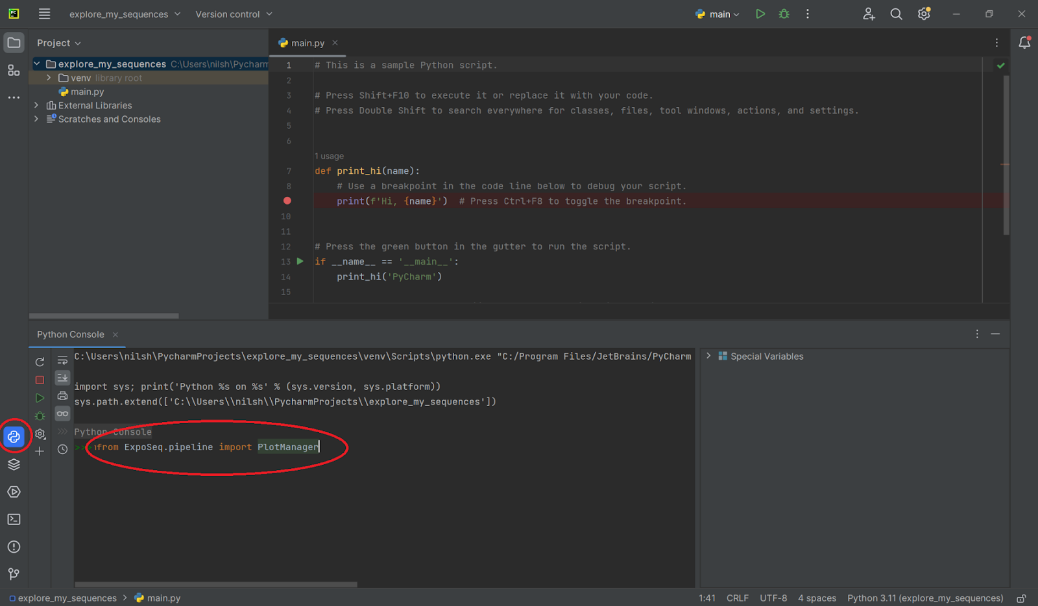


Step 5: Initialize the PlotManager with *plot = PlotManager()* and answer the questions which will be prompted subsequently:


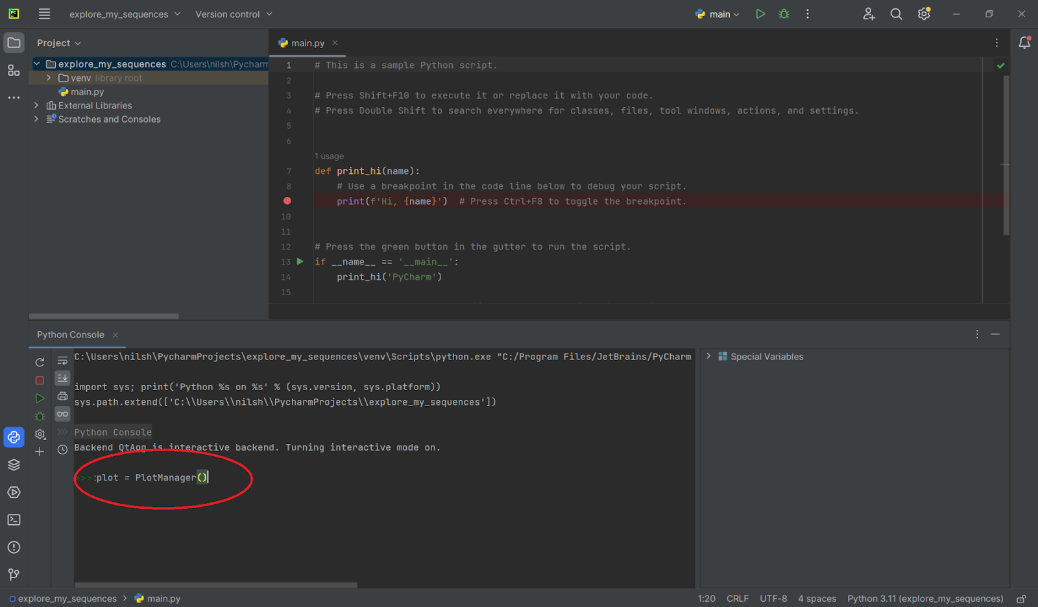


Step 6: Choose the correct upload option. You will be asked to either upload a new experiment or choose an existing experiment. If you want to upload a new experiment and analyse this using MiXCR, press 1. If you instead want to continue with an earlier experiment, or if you have mixcr data analyzed elsewhere (i.e. on a server for larger datasets) you can choose option 2 to upload this.


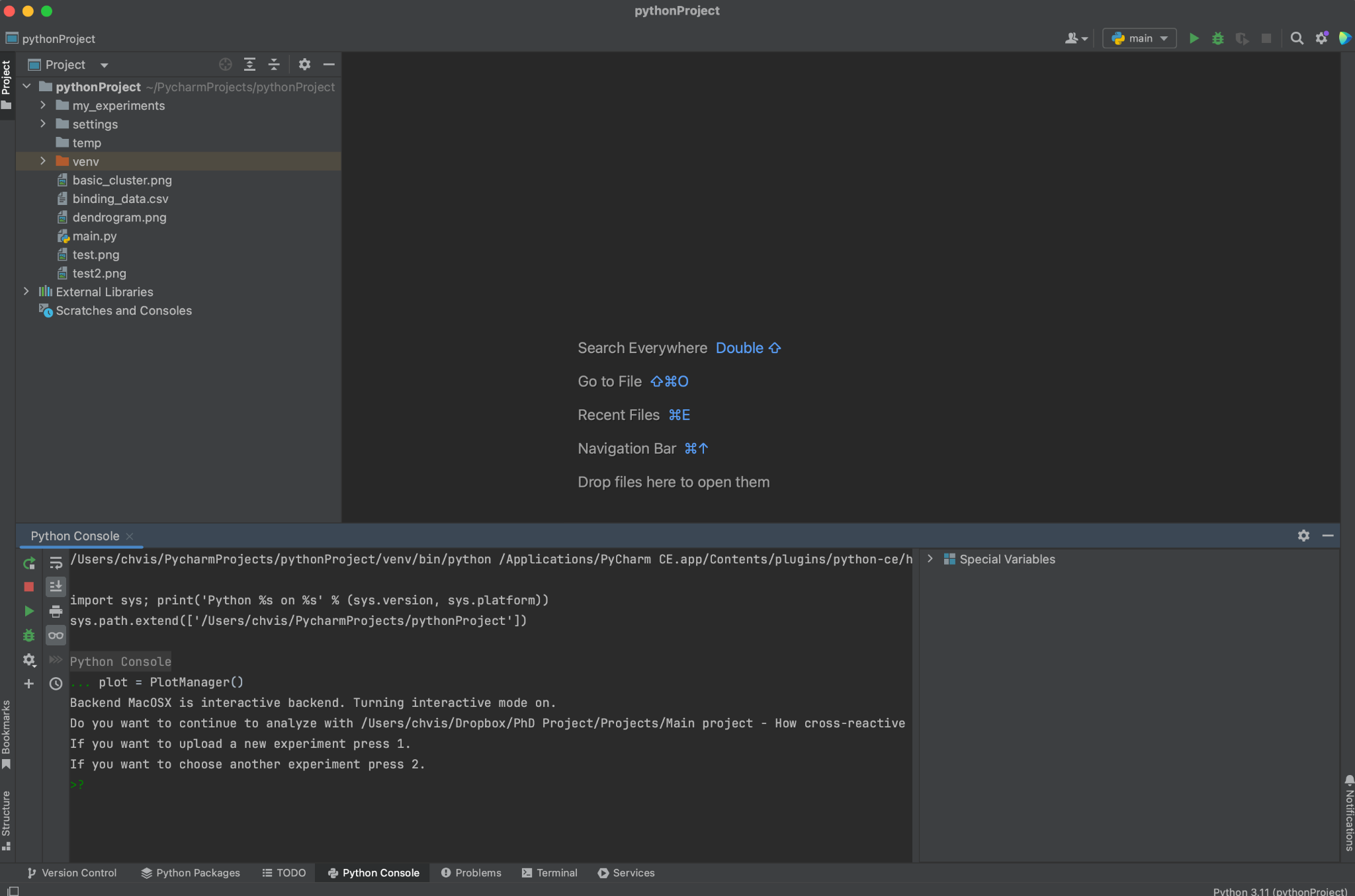


With this option, the pipeline should guide you through the initial steps and then start analyzing your data and generate all plots. OBS: The MiXCR step can take a long time - for the dataset used to build this, the MiXCR step takes several hours - but it only has to be done once.

In case the pipeline does not automatically generate your plots, or you have picked option 2 in the beginning, you can initiate the pipeline by typing plot.full_analysis().

It is also possible to generate each plot manually and modify it to fit your needs using the different plots which pops up when typing plot. and then hitting the tab button. You can follow this [interactive script](https://github.com/nilshof01/ExpoSeq/blob/final_master/ExpoSeq_handsOn.ipynb) where you can see how you can create specific plots. Importantly, if you are unsure what you need to give as input for certain figures, you can use the help() command. For example, if you  would like to create a distribution of the HCDR3 lengths in your sample then you can type the following:
 *help(plot.length_distribution_single)*

## Supplementary Figures

##
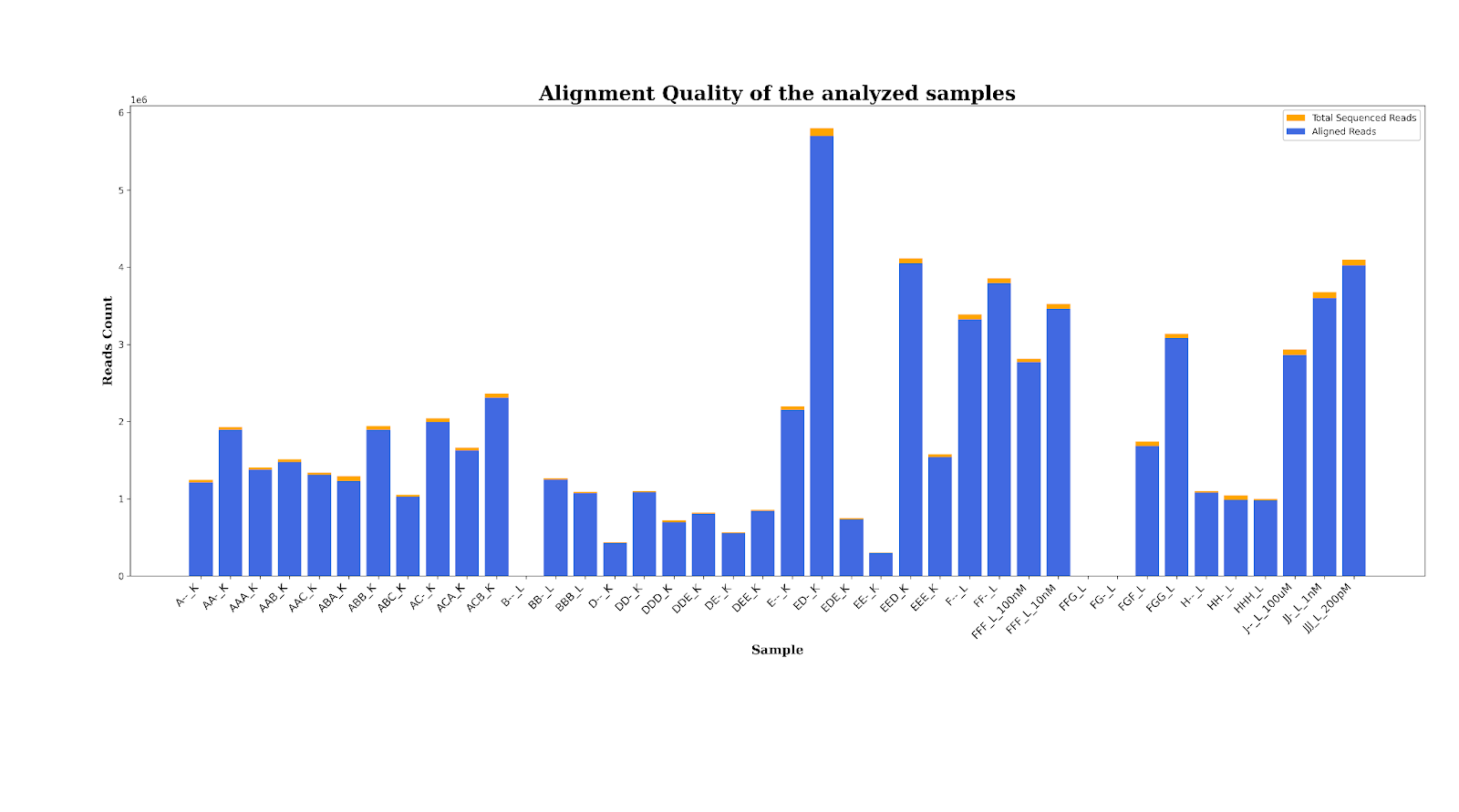


**Supplementary Fig. S1: Alignment Quality.** More information with: help(plot.alignment_quality)


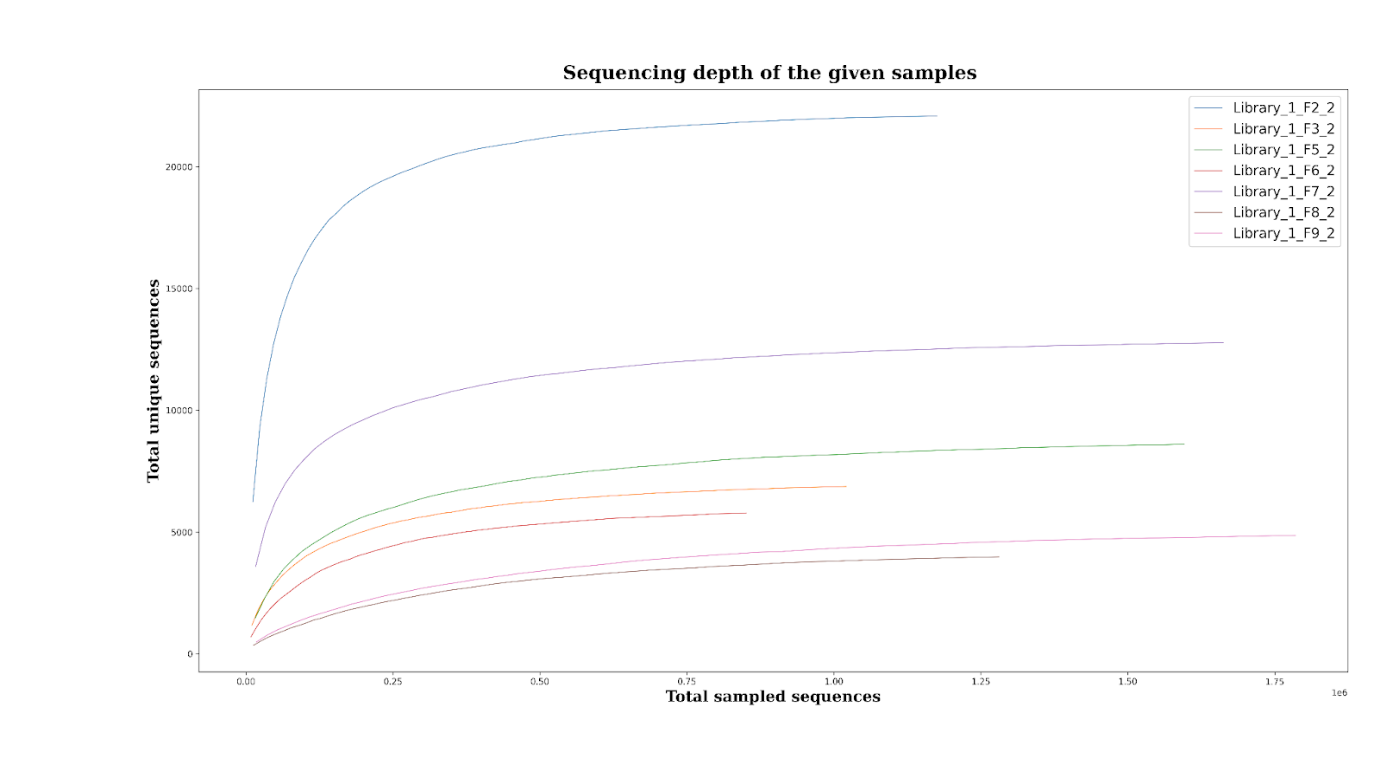
 **Supplementary Fig. S2.  Rarefaction curves.** More information with: help(plot.rarefraction_curves)


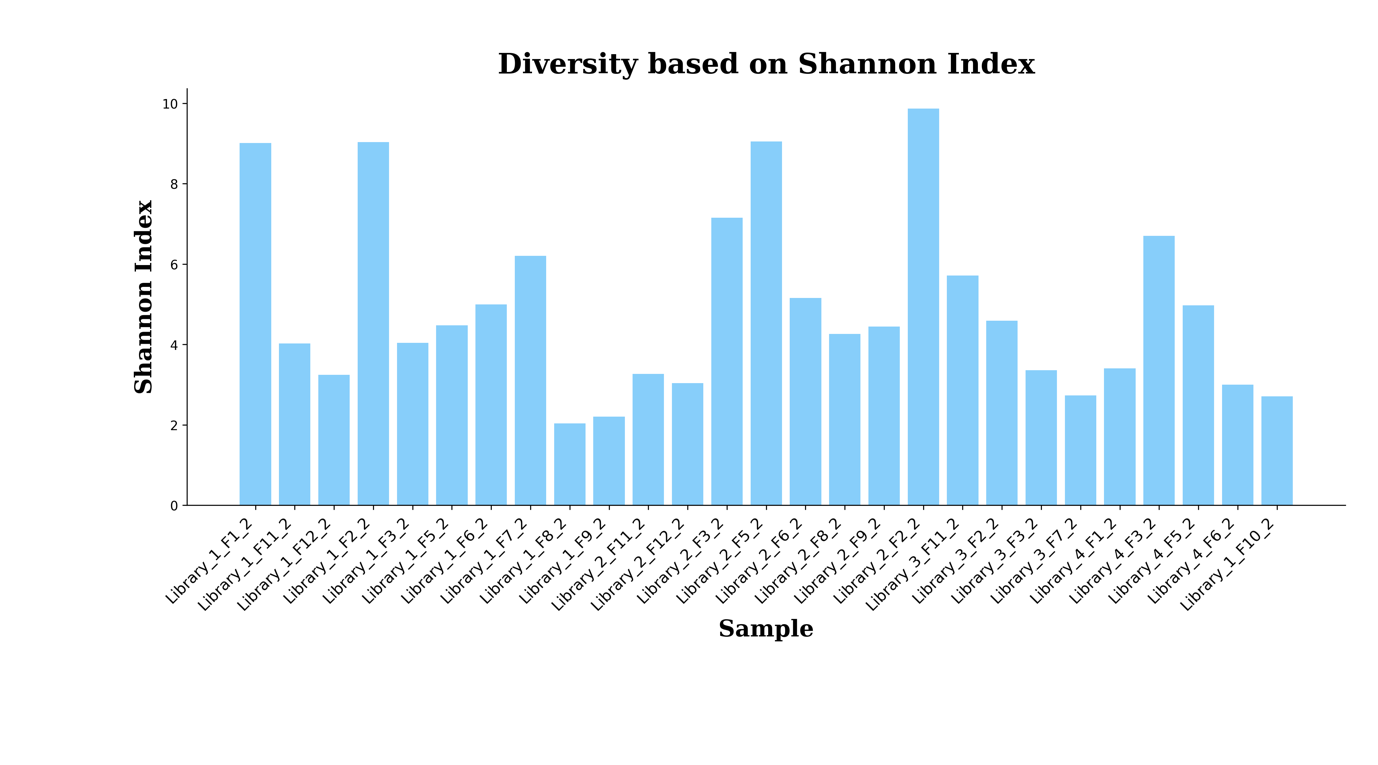


**Supplementary Fig. S3.  Diversity based on Shannon index.** Plot can be generated with plot.sample_diversity("Shannon").


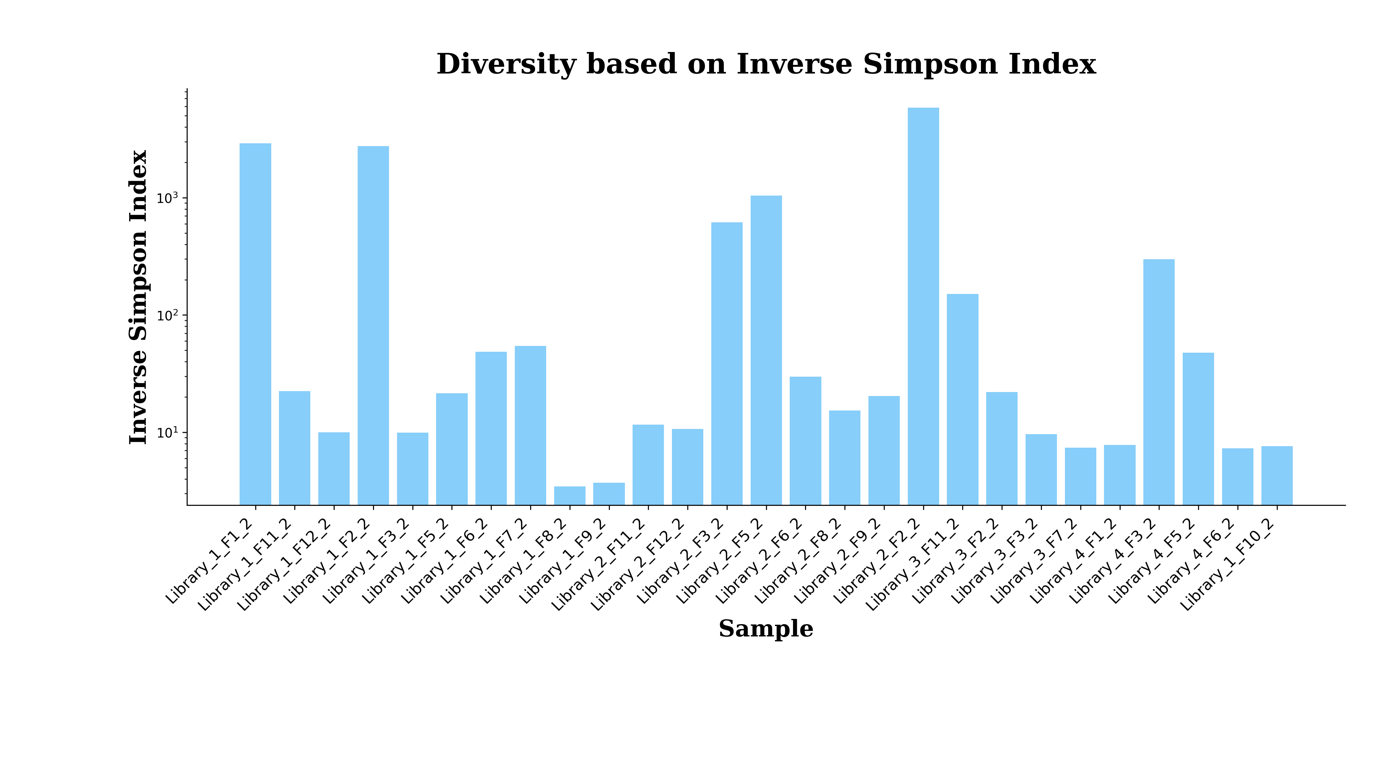


**Supplementary Fig. S4.  Diversity based on Inverse Simpson index.** Plot can be generated with plot.sample_diversity().


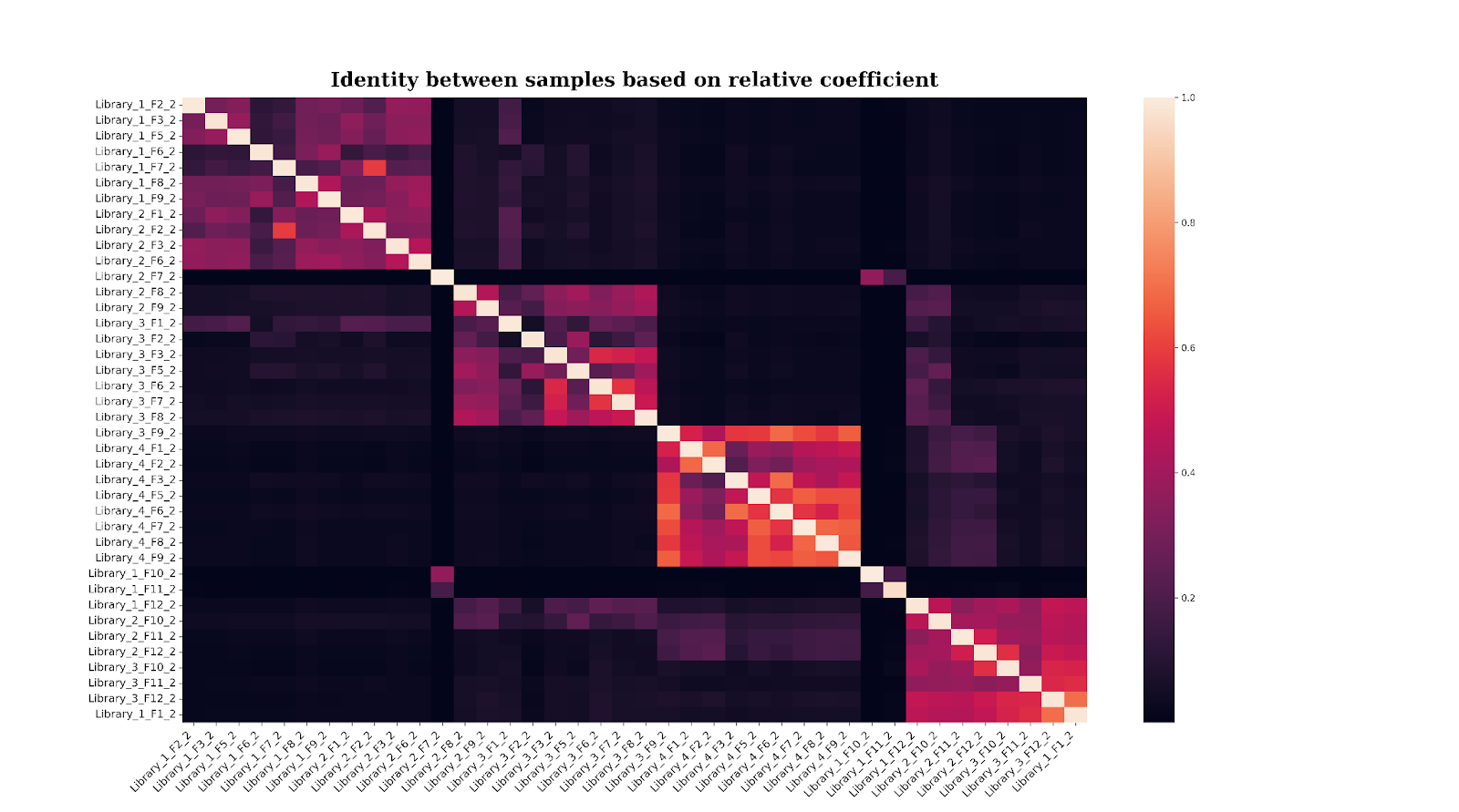


**Supplementary Fig. S5. Heatmap showing the identity based on relative counts of reads between samples.** More information with: help(plot.relative)


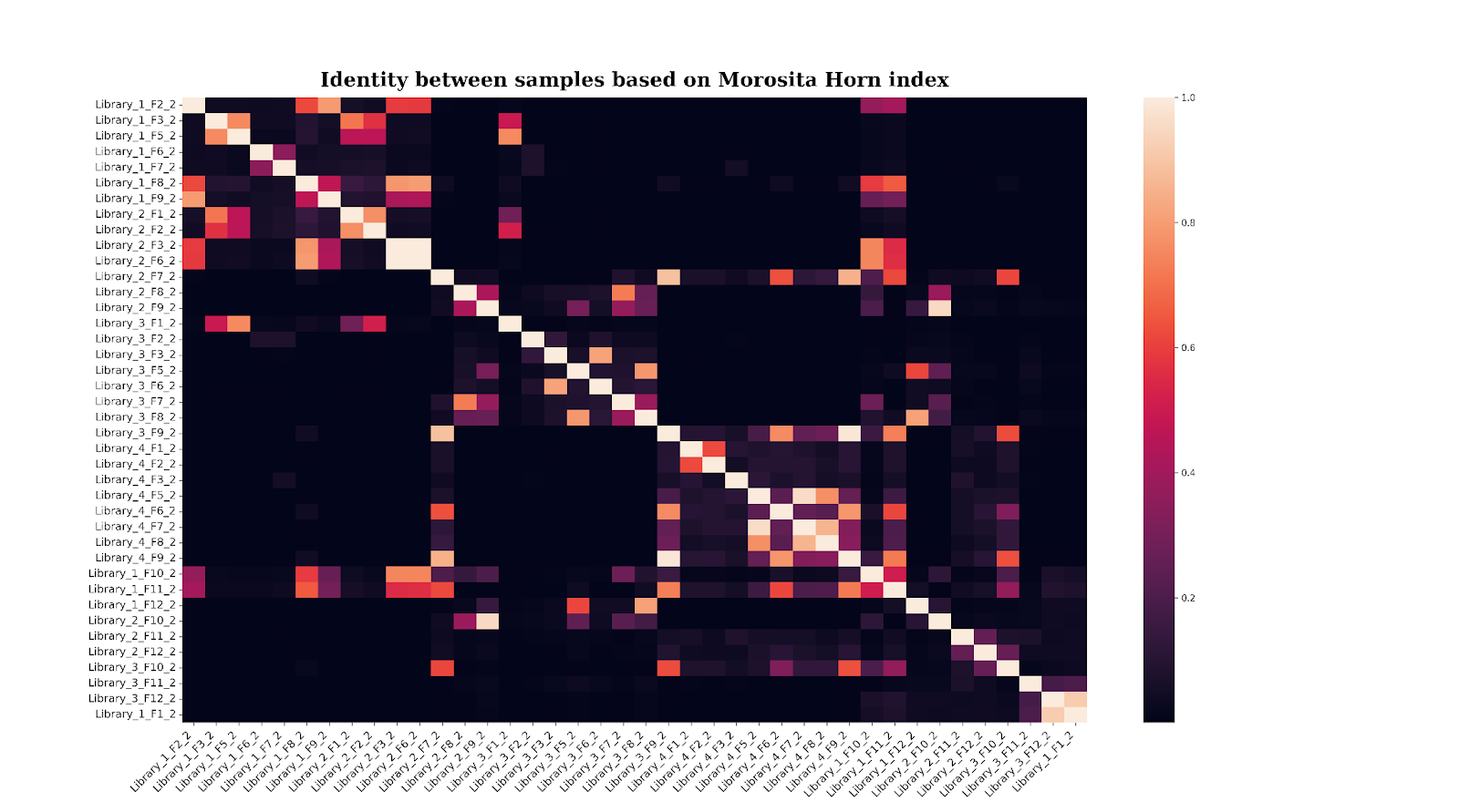


**Supplementary Fig. S6. Heatmap showing the identity based on Morosita-Horn index.** More information with: help(plot.morosita_horn)


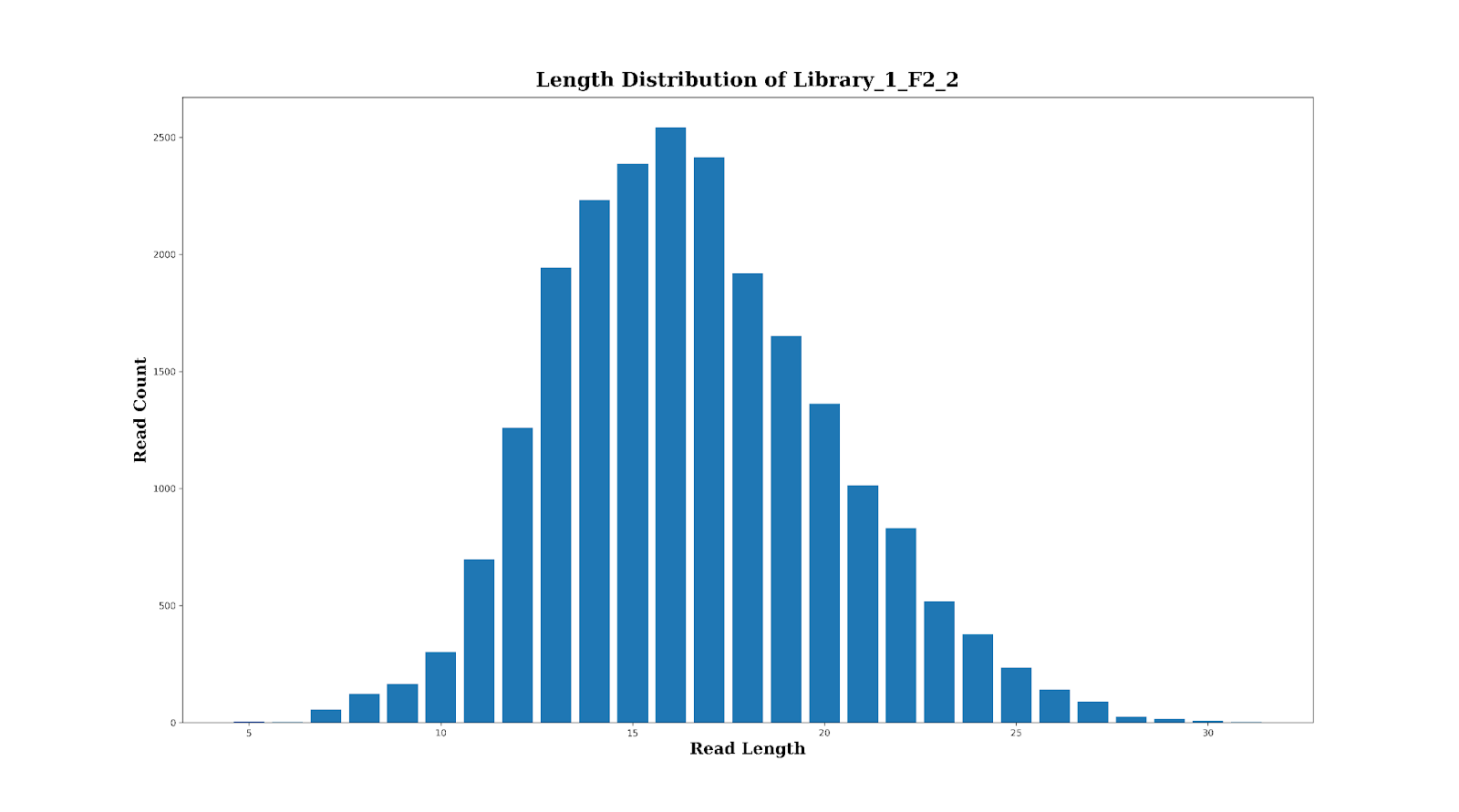


**Supplementary Fig. S7. Length Distribution of reads in the given sample.** More information with: help(plot.length_distribution_single)


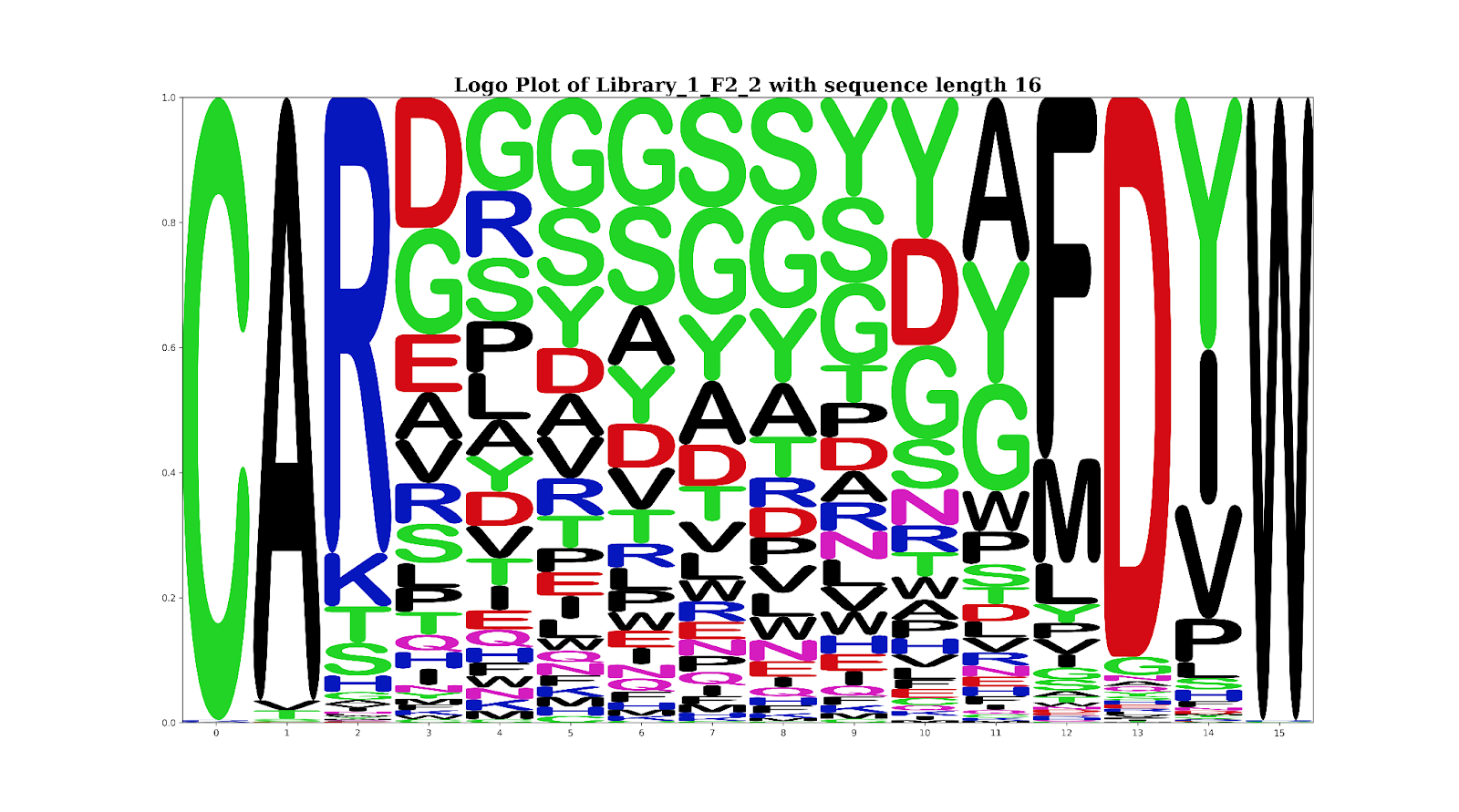


**Supplementary Fig. S8. Logo plot.** More information with: help(plot.logoPlot_single)


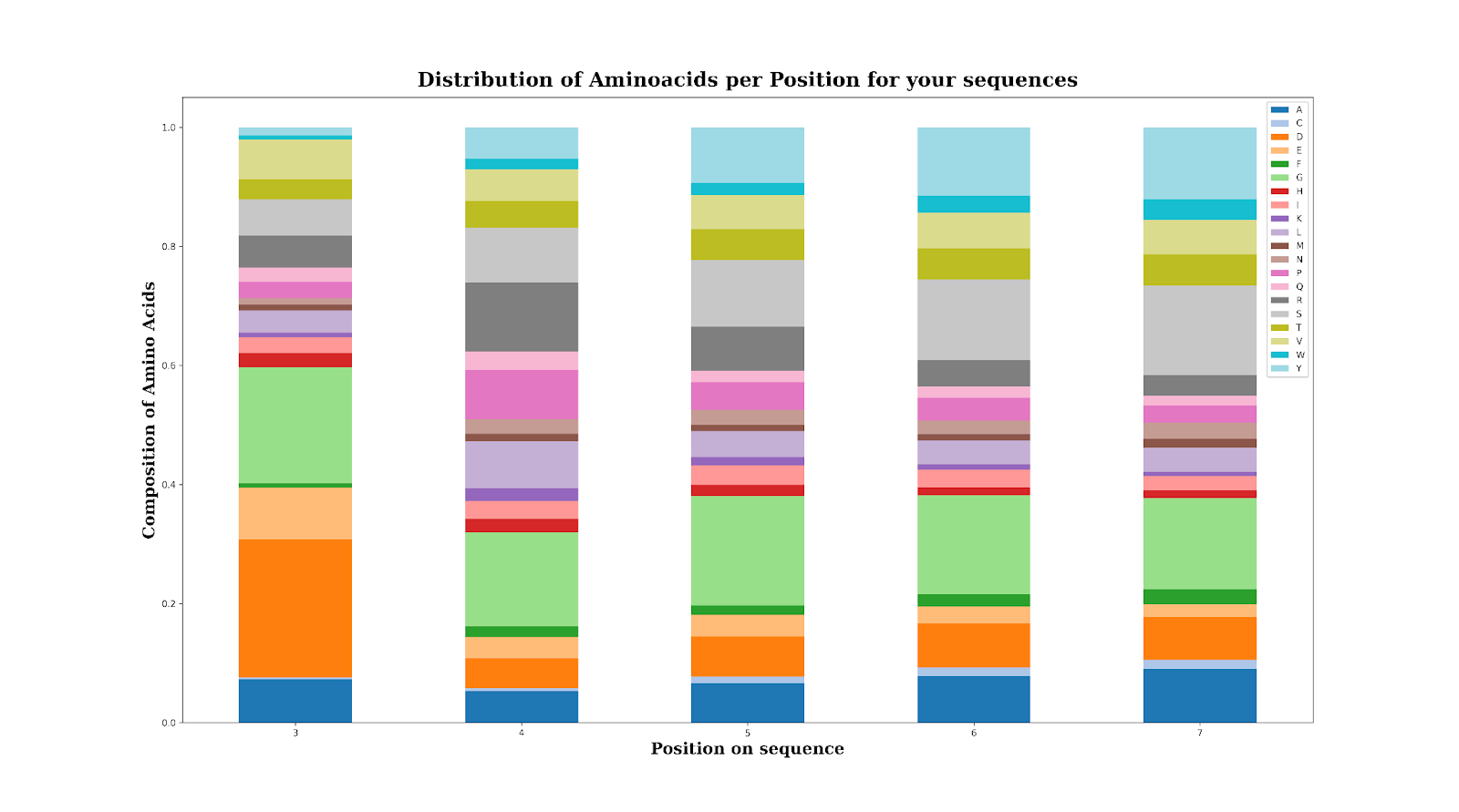


**Supplementary Fig. S9. Amino acid distribution for a specific region.** More information with: help(plot.aa_distribution)


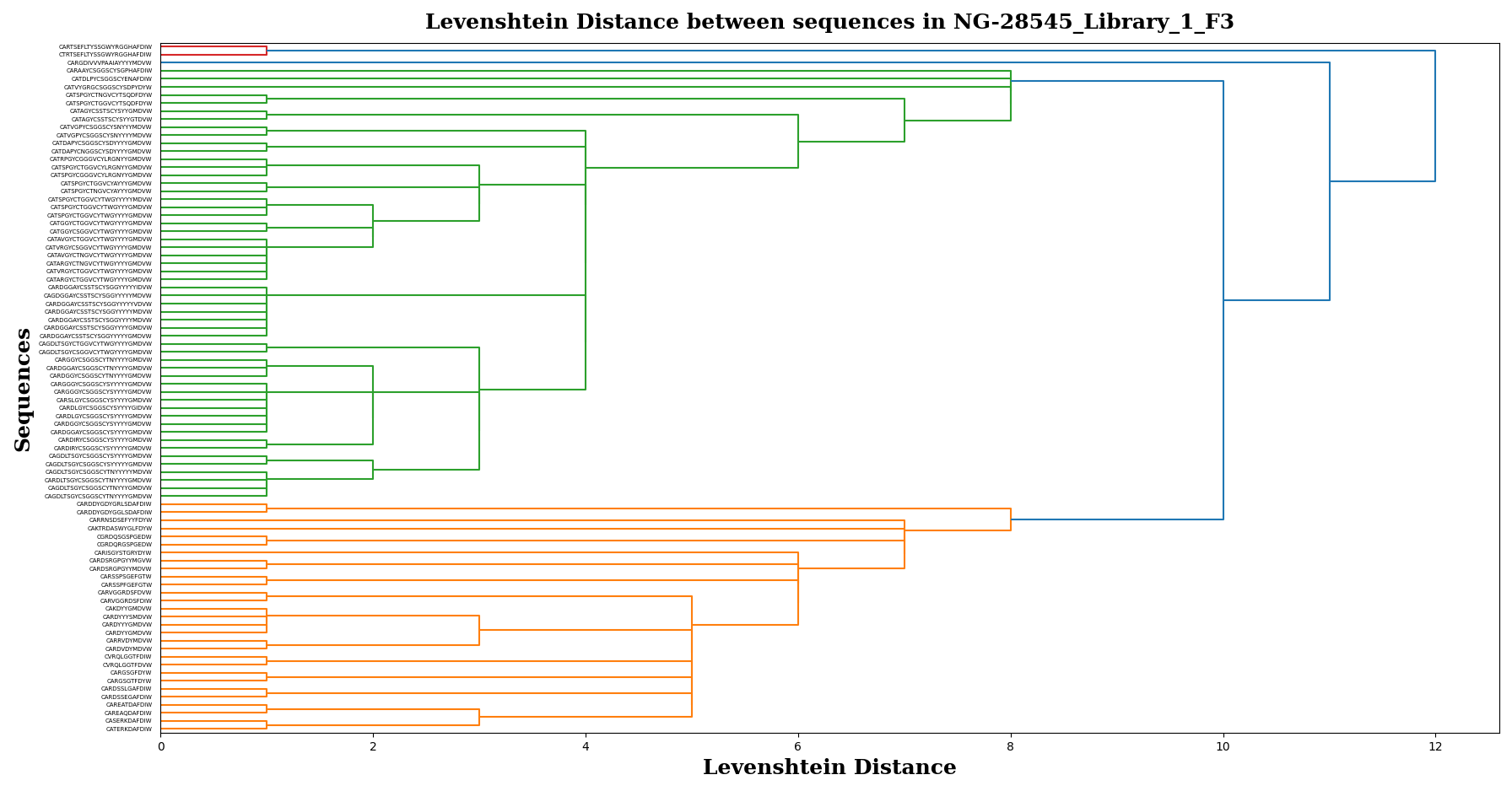


**Supplementary Fig. S10. Dendrogram using levenshtein distance using a batch size of 1000 and the default max cluster size.** More information with: help(plot.levenshtein_dendrogram)

**
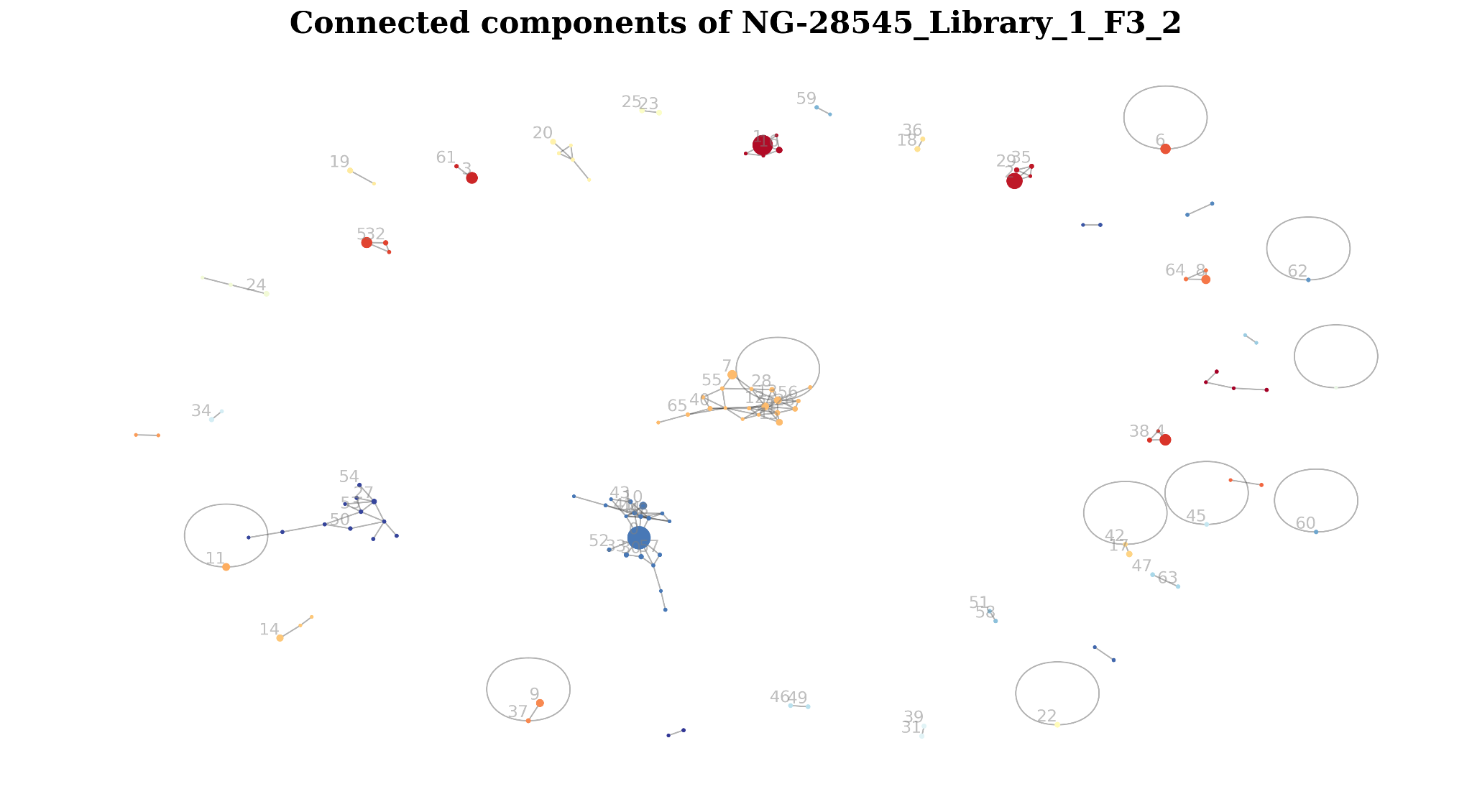
**

**Supplementary Fig. S11. Clustering showing all sequences that have an LD of maximum 1.** Arrows between two nodes mean they have an LD of 1. Positioning and lengths of arrows is irrelevant. More information with: help(plot.basic_cluster)


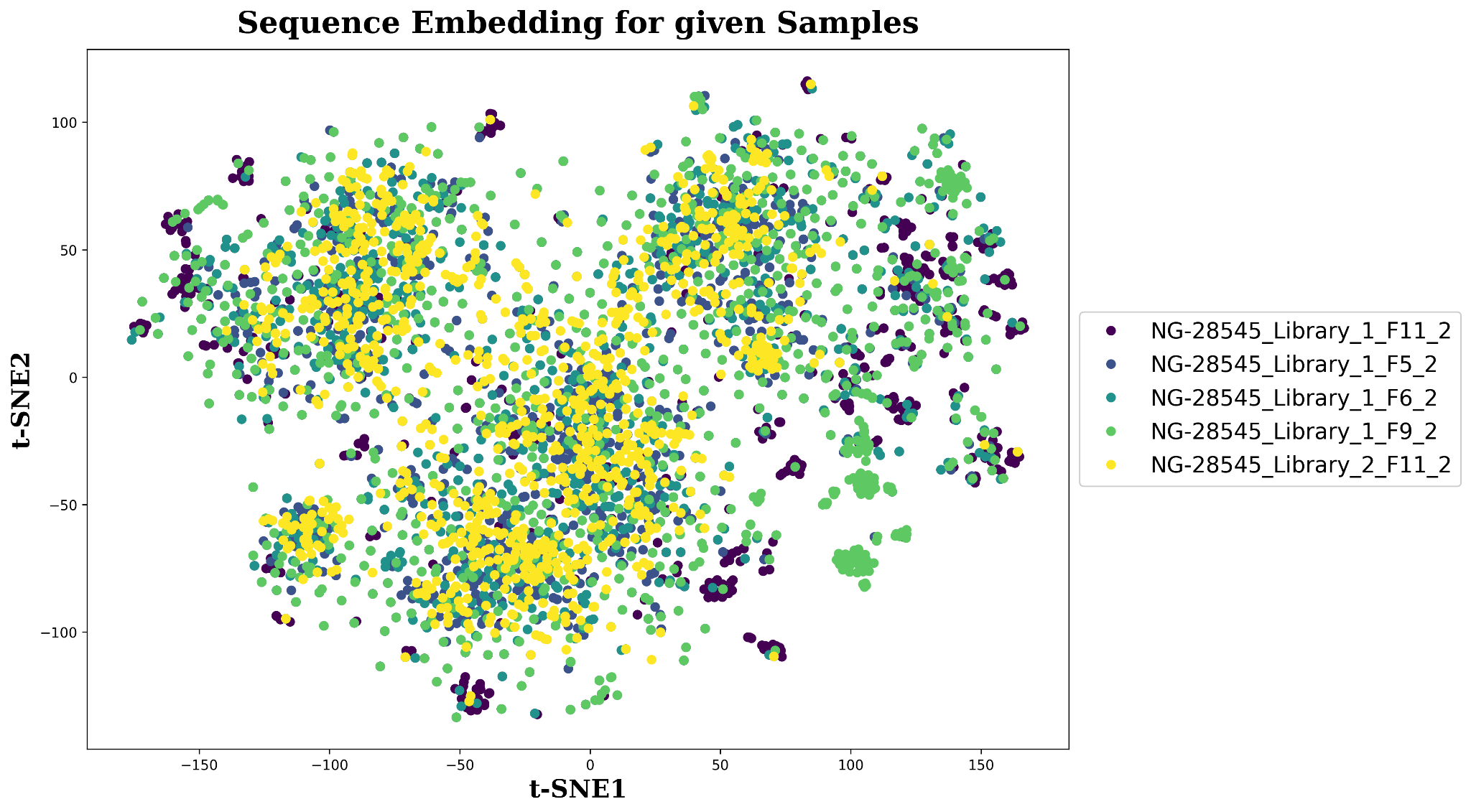


**Supplementary Fig. S12: Sequence embedding of three samples**. More information with: help(plot.embedding_tsne)


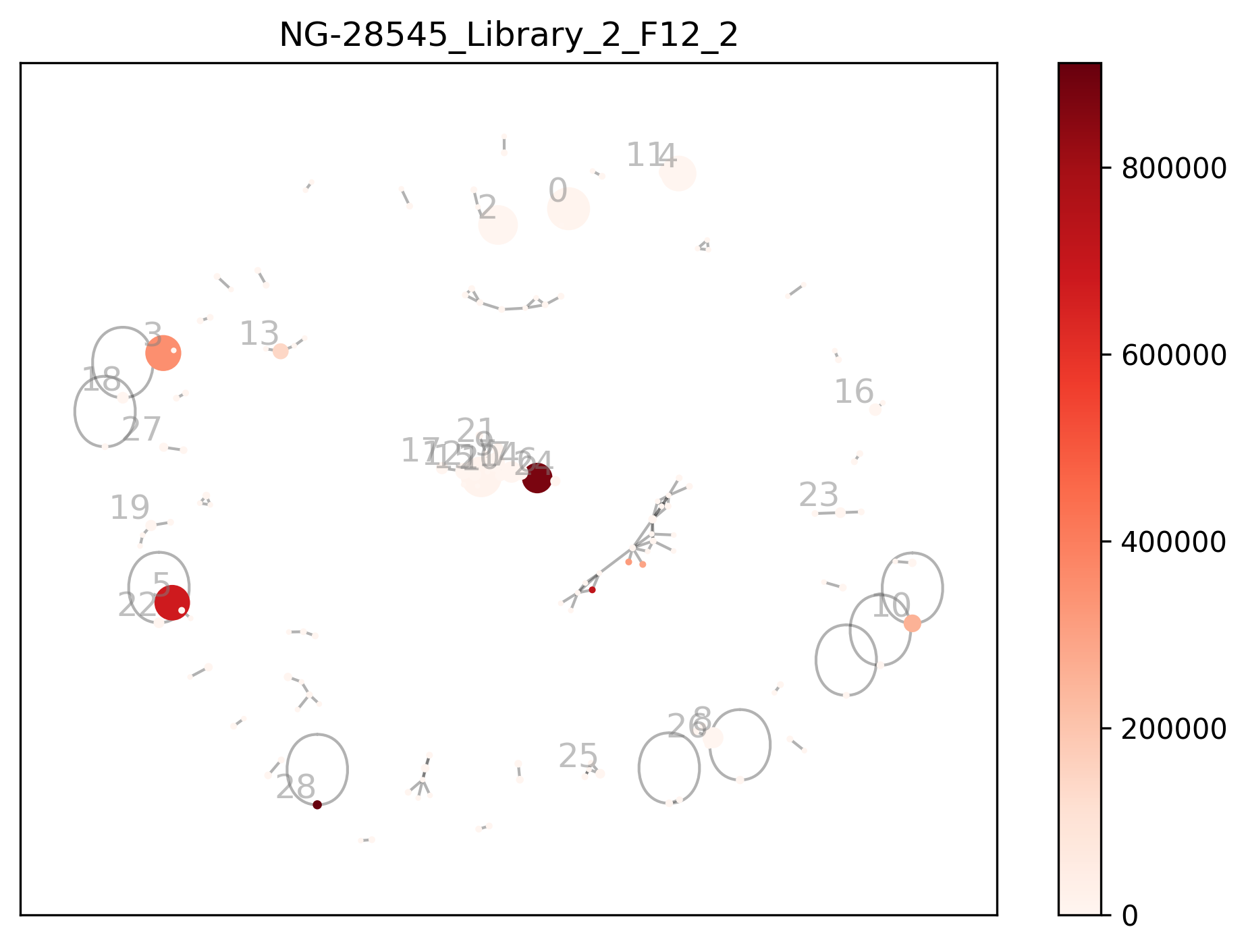


**Supplementary Fig. S13: Clustering of sequences in sample NG-28545_Library_2_F3_2 with a maximum LD of 1.** Node size represents sequence abundance and color bar represents binding values. Connected nodes have an LD of 1. Positioning of nodes is irrelevant. Self-connecting (the circular connections) nodes are irrelevant connections and is simply an artefact of the script used to generate the plot. More information with: help(plot.cluster_one_AG)


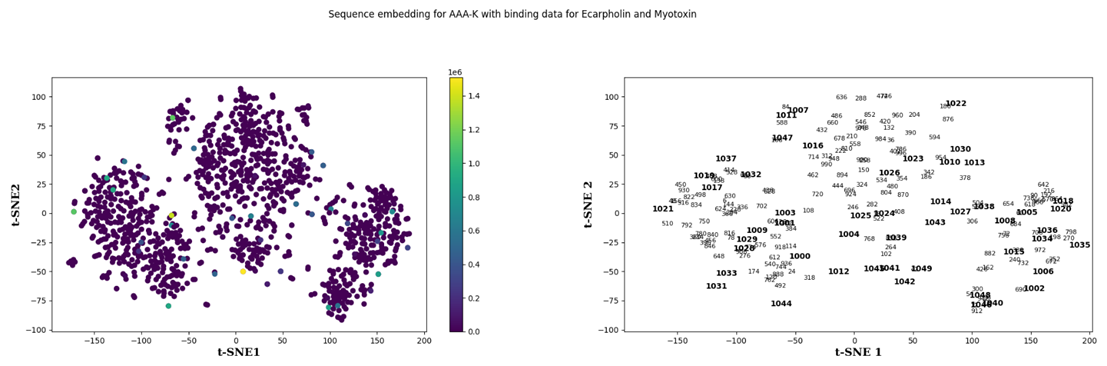


**Supplementary Fig. S14: Sequence embedding of panning round with sequences from sanger sequencing.** Sanger sequences are labelled with corresponding binding data in t-SNE space together with NGS data - more information with: help(plot.tsne_cluster_AG)

## How to add the binding data correctly

ExpoSeq allows users to choose whether they would like to integrate binding data with corresponding sequences or not. While the pipeline offers a wide range of figures without binding data, users also have an option to introduce their binding data. For instance, they can import one or multiple csv (comma separated values) files with the sequences and the binding data for different epitopes from sanger sequencing. It is important to provide the data in the correct format, so that the pipeline can integrate the data appropriately. You can create the file in excel and export it as csv but the header should be identical to the first row in the given image:


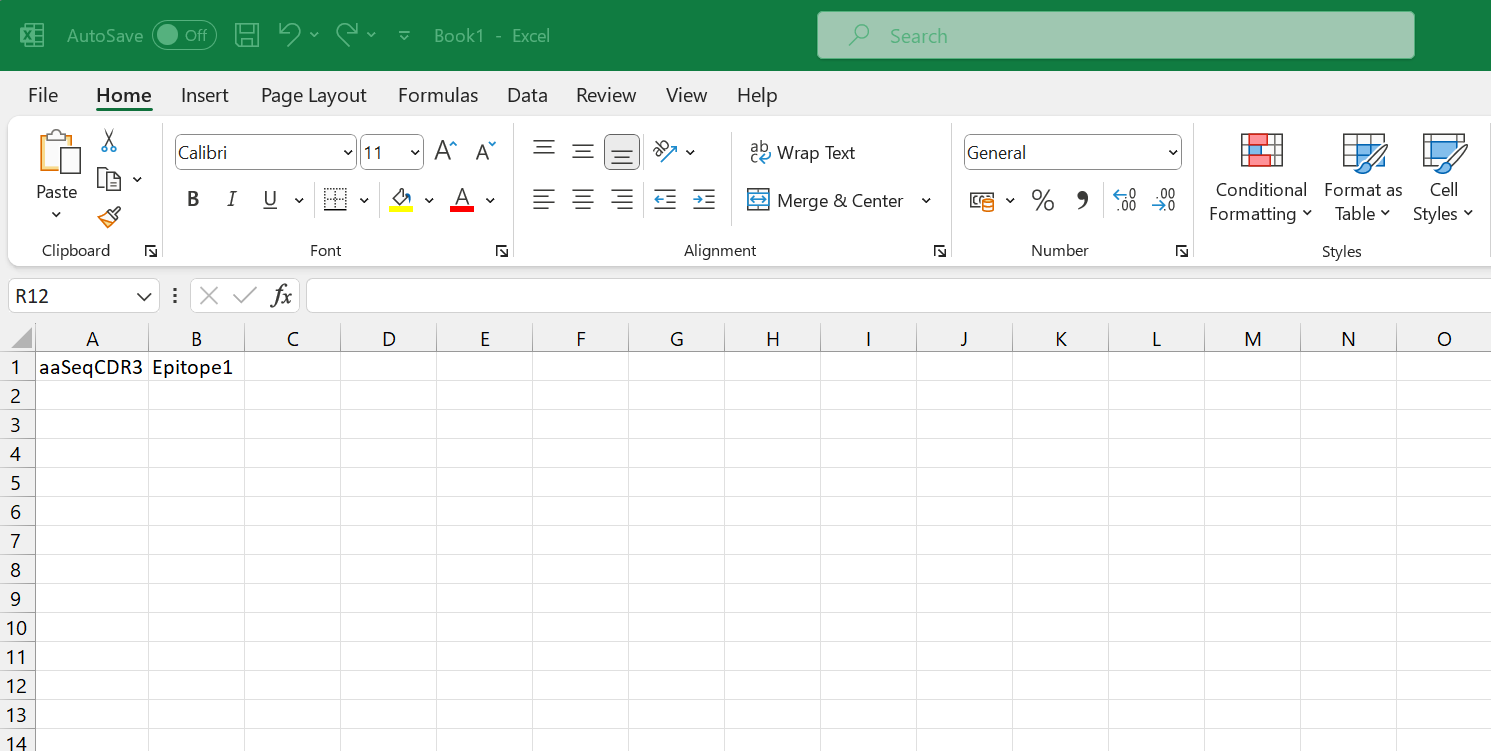


You can change the name for Epitope1 but aaSeqCDR3 needs to remain the same. Subsequently you can insert the sequences and the values from the binding assays you have carried out, i.e. ELISA values.

## Automation of reports and interactive dashboards

After the pipeline has finished the automatic generation of the visualizations from your dataset, the users can have a deeper and more user-friendly insight by creating an interactive dashboard to visualize base parameters of your data and automatically create a report as a summary of the generated plots. The report contains additional material about the plots, including a description of each of them and how they may be interpreted. Further the report can easily be customized by using the .qmd file which is generated by the software Quarto. Thus, this software has to be installed before this feature can be used.

## Reproducibility

ExpoSeq has been developed in PyCharm using Python 3.11 on Windows 11. It also works in Python 3.8, 3.9 and 3.10. The package can be installed via pip and all its dependencies are automatically downloaded during the installation. The pipeline can be tested using the test_version of ExpoSeq where the sequencing report and the binding data is automatically sampled using the script randomizer which can be found in the folder augment_data. All software tools and dependencies for version [ExpoSeq 4.2.1](https://pypi.org/project/ExpoSeq/) are summarized in the following table.

| **Programming Language** | **Version** |
| --- | --- |
| Python | 3.11 |
| **Software** | **Version** |
| Windows | 11 Home (22H2) |
| PyCharm | 2022.3.2 |
| MiXCR | 4.2.0 |
| Quarto | 1.3.450 |
| **Packages** | **Version** |
| numpy | 1.24.1 |
| pandas | 1.5.3 |
| matplotlib | 3.6.3 |
| scipy | 1.10.0 |
| seaborn | 0.12.2 |
| logomaker | 0.8 |
| editdistance | 0.6.2 |
| networkx | 2.6.3 |
| PyQt5 | 5.15.8 |
| scikit-learn | 1.2.1 |
| openpyxl | 3.1.2 |
| python-louvain | 0.16 |
| pandasai | 1.3.3 |
| markdown | 3.4.4 |
| sweetviz | 2.2.1 |
| ydata-profiling | 4.6.0 |

## Contact and feedback

For any kind of feedback or further ideas please contact Nils Hofmann via email at nilhof01@gmail.com. Furthermore, please feel invited to start a discussion or discuss with other researchers about new findings in antibody discovery on [ExpoSeq’s repository](https://github.com/nilshof01/ExpoSeq/discussions).
